# Detection of Cervical Cancer Biomarker Patterns in Blood Plasma and Urine by Differential Scanning Calorimetry and Mass Spectrometry

**DOI:** 10.1371/journal.pone.0084710

**Published:** 2014-01-08

**Authors:** Nichola C. Garbett, Michael L. Merchant, C. William Helm, Alfred B. Jenson, Jon B. Klein, Jonathan B. Chaires

**Affiliations:** 1 James Graham Brown Cancer Center, University of Louisville, Louisville, Kentucky, United States of America; 2 Department of Medicine, University of Louisville, Louisville, Kentucky, United States of America; 3 Center for Environmental Genomics and Integrative Biology, University of Louisville, Louisville, Kentucky, United States of America; 4 Division of Gynecologic Oncology, University of Louisville, Louisville, Kentucky, United States of America; 5 Robley Rex Veterans Affairs Medical Center, Louisville, Kentucky, United States of America; Universidad de Granada, Spain

## Abstract

Improved methods for the accurate identification of both the presence and severity of cervical intraepithelial neoplasia (CIN) and extent of spread of invasive carcinomas of the cervix (IC) are needed. Differential scanning calorimetry (DSC) has recently been shown to detect specific changes in the thermal behavior of blood plasma proteins in several diseases. This methodology is being explored to provide a complementary approach for screening of cervical disease. The present study evaluated the utility of DSC in differentiating between healthy controls, increasing severity of CIN and early and advanced IC. Significant discrimination was apparent relative to the extent of disease with no clear effect of demographic factors such as age, ethnicity, smoking status and parity. Of most clinical relevance, there was strong differentiation of CIN from healthy controls and IC, and amongst patients with IC between FIGO Stage I and advanced cancer. The observed disease-specific changes in DSC profiles (thermograms) were hypothesized to reflect differential expression of disease biomarkers that subsequently bound to and affected the thermal behavior of the most abundant plasma proteins. The effect of interacting biomarkers can be inferred from the modulation of thermograms but cannot be directly identified by DSC. To investigate the nature of the proposed interactions, mass spectrometry (MS) analyses were employed. Quantitative assessment of the low molecular weight protein fragments of plasma and urine samples revealed a small list of peptides whose abundance was correlated with the extent of cervical disease, with the most striking plasma peptidome data supporting the interactome theory of peptide portioning to abundant plasma proteins. The combined DSC and MS approach in this study was successful in identifying unique biomarker signatures for cervical cancer and demonstrated the utility of DSC plasma profiles as a complementary diagnostic tool to evaluate cervical cancer health.

## Introduction

Invasive carcinoma of the uterine cervix (IC) is the third most common cancer affecting women with an estimated 529,000 cases diagnosed worldwide in 2008 and 274,000 deaths [1]. In recent decades routine screening has helped to significantly reduce both the incidence and deaths from IC in the USA but, nevertheless, an estimated 12,340 new cases will be diagnosed with 4,030 deaths in 2013 [2]. Invasive cervical cancer is preceded by a precancerous condition, cervical intraepithelial neoplasia (CIN), in which abnormal cell growth occurs in the epithelial lining of the cervix. CIN is divided into grades (1 = mild, 2 = moderate, 3 = severe) based on histologic features including nuclear changes and the extent of involvement of the epithelium. The risk for progression of CIN to IC over time rises by grade, being highest for CIN 3 [3], [4]. CIN can be treated to greatly reduce the chance of IC developing. Partly to help with treatment planning, CIN 2 and CIN 3 are commonly grouped together as high-grade squamous intraepithelial lesion (HSIL) which would be treated and CIN 1 and HPV condyloma as low-grade squamous intraepithelial lesion (LSIL) which would be untreated. Once IC is detected the principal need is to determine whether there is early stage disease (FIGO Stage I) which is confined to the cervix or if there is more advanced metastatic disease. Only disease confined to the cervix and of sufficiently small size is considered treatable with surgery.

Reliable methods to accurately detect CIN and IC are critical. Current screening cannot differentiate low-grade from higher grade CIN, CIN from IC, early from more advanced stages of IC, or determine additional disease status indicators such as the presence of nodal involvement. For many years the initial screening method for CIN has been the Pap smear test which enables cytologic abnormalities to be detected on scrapings from the cervix. Women with Pap smears suggesting squamous intraepithelial lesions would then be evaluated, including by clinical examination and colposcopy and biopsy, to determine the grade and extent of any CIN present and to exclude (or diagnose) the presence of IC. Pap smear screening is now being integrated with testing for high-risk genotypes of human papilloma virus (HPV HR) which can be detected in the same cytologic Pap smear sample using liquid-based Hybrid Capture II technology [5]. All grades of CIN are associated with a high likelihood of the presence of HPV HR. In women between 30 and 65 years HPV HR testing improves the detection rate of CIN 3 or greater by 17–31% in the first round of screening and reduces the incidence of IC in the second round of screening [6], [7], [8], [9]. HPV HR testing is now also being integrated into the follow-up of women who have previously been shown to have HPV HR infection or CIN, since persistent HPV HR infection is associated with an increased risk for the development of recurrent CIN and IC [6], [10]. Although testing for HPV HR can improve the detection of CIN it cannot differentiate between CIN lesions having a higher likelihood of progressing from those that do not. Biomarkers have been investigated in this regard but have not yet proven useful [11], [12], [13], [14]. Work-up and treatment for women with abnormal Pap smears is traumatic physically, emotionally and financially and, unfortunately, because of the inability to differentiate CIN lesions having a higher likelihood of progression to IC many women still get over treated.

Here we describe the application of a combined approach using differential scanning calorimetry (DSC) and mass spectrometry (MS) for the characterization of cervical disease. DSC is commonly used to measure the denaturation profiles of biomolecules, known as thermograms, by directly monitoring the heat changes associated with molecular events as a function of temperature. Under given solution conditions, a thermogram provides a unique signature for a given protein reflecting specific structural motifs and molecular forces. It has recently been shown that DSC can be applied to the analysis of blood plasma to provide an indication of an individual’s clinical status [15], [16], [17], [18], [19], [20], [21], [22], [23], [24]. The thermogram of plasma from healthy individuals reflected the weighted sum of the denaturation profiles of the most abundant plasma proteins; however, thermograms from individuals with various conditions or diseases were significantly changed [15], [16], [17], [18]. Thermogram changes appeared to be sensitive to the particular disease state and indicated that plasma DSC might have utility as a clinical diagnostic screening method. Mechanisms for disease-specific changes in thermograms are currently under investigation in our laboratory but we hypothesize that they reflect the presence of disease biomarkers that modify or interact with plasma proteins thereby affecting their denaturation properties. Such biomarker interactions would support the “interactome” concept that proposes that low molecular weight peptides present in the plasma proteome are complexed with more abundant plasma proteins creating a network of protein-protein and peptide-protein interactions [25]. Although the presence of putative low molecular weight biomarkers can be inferred through changes in the thermogram no direct identification can be made by this method. We have applied a MS peptidomic approach to investigate the nature of plasma thermogram profiles associated with cervical disease.

MS has been used as a tool to aid in the understanding of disease pathogenesis and the generation of candidate biomarkers of disease [26], [27], [28], [29], [30], [31], [32]. One application of MS has been the quantitative and qualitative analysis of low molecular weight protein fragments found in plasma and/or urine, referred to as a peptidomic approach [33], with the goal to correlate abundance with detection or prognosis of disease. The peptides observed in these studies are reflective of the metabolic turnover of the parent proteins with abundance changes between case (disease) and control conditions related to an alteration(s) to the protein’s normal catabolic or anabolic metabolic half-life. These peptidomic studies have in some cases successfully demonstrated correlations between amounts or patterns of several peptides with the disease or the disease progression. With respect to cervical disease, the metastatic potential of CIN has been correlated to the expression of three proteins including matrix metalloproteinase-2 (MMP-2), MMP-9, and urokinase-type plasminogen activator [14]. Because of the primary involvement of peptide generating metalloproteinases in the disease progression, this correlation of CIN could be extended to the peptidomic phenotype. As such, the presence of unique plasma and/or urinary peptide profile(s) could be established by MS approaches and be correlated with thermogram profiles reflecting the presence and extent of cervical disease. We describe results demonstrating sensitivity of DSC thermograms to clinical status and evidence for the association of thermogram profiles with interacting peptide biomarkers identified by MS.

## Materials and Methods

### Specimen Collection

The study protocol and patient consent procedures were approved by the University of Louisville Institutional Review Board (IRB# 08.0108, 608.03). Women attending the clinics of the Division of Gynecologic Oncology with biopsy-proven cervical intra-epithelial neoplasia and invasive cervical carcinoma were eligible and gave written informed consent for their blood and tissues to be entered into a tissue repository (IRB# 608.03) and utilized for research purposes. The IRB specifically approved the use of tissues from the tissue repository for use in this study without the need for further consent (IRB# 08.0108). Women known to be HIV positive were ineligible. Blood and spot urine samples were collected from recruited patients during their initial clinic visit at the time of evaluation and before receiving therapy. Urine samples were aliquoted and immediately stored at −80°C until analysis. Blood was drawn into 5 mL purple top (plasma; K_2_-EDTA anticoagulant) vacutainers. Tubes were gently mixed by inversion 8–10 times immediately after blood collection to evenly distribute the anticoagulant additive followed by centrifugation at 3200 rpm for 10 minutes (BD-Clay Adams Compact II centrifuge). Separated plasma was carefully aspirated to avoid hemolysis or contamination of the separated blood phases, aliquoted and immediately stored at −80°C until analysis. Control blood and urine specimens were collected under the same study procedures from healthy volunteers with no history of abnormal Pap smears or cancer. As above, the study protocol and consent procedures were approved by the University of Louisville Institutional Review Board (IRB# 08.0108, 608.03). All volunteers gave written, informed consent for their blood and tissues to be entered into a tissue repository (IRB# 608.03) and utilized for research purposes. The IRB specifically approved the use of tissues from the tissue repository (IRB# 608.03) for use in this study without the need for further consent (IRB# 08.0108). Specimen aliquots were thawed for analysis and the remainder discarded. All handling of specimens and specimen waste was in accordance with OSHA bloodborne pathogen procedures. All specimens collected for the study were deidentified and stored in the Gynecologic Oncology Tissue Bank at the James Graham Brown Cancer Center. Associated demographic and clinical information was collected by clinical study personnel and securely stored on the study computer. The tissue bank was approved by the University of Louisville Institutional Review Board (IRB# 608.03) and was fully HIPAA compliant. Specimens provided to basic science study personnel (NCG, MLM, ABJ, JBK, JBC) for DSC and MS studies were coded by tissue bank collection number. In this form specimens were deidentified and blinded for both demographic and pathologic disease status for unbiased data collection. Demographic and disease status was subsequently provided for data sorting and interpretation.

### DSC Studies

#### Sample preparation for DSC studies

Plasma samples (100 µL) were dialyzed against a standard phosphate buffer (1.7 mM KH_2_PO_4_, 8.3 mM K_2_HPO_4_, 150 mM NaCl, 15 mM sodium citrate, pH 7.5) for 24 hours at 4°C in order to achieve normalization of buffer conditions for all samples. Frozen plasma samples were thawed overnight at 4°C the day before dialysis. On the morning of dialysis, samples were loaded into Slide-A-Lyzer mini dialysis units (MWCO 3,500; Pierce, Rockford, IL) that had been equilibrated against dialysis buffer overnight. Based on cost and reliability we routinely re-assembled washed dialysis units replacing the original dialysis membrane with cut-to-size Snakeskin Pleated Dialysis Tubing (Pierce, Rockford, IL). Samples were dialyzed against 1 L of phosphate buffer with buffer changes after three hours of dialysis, then after two periods of four hours with a final overnight dialysis period. Samples were recovered from dialysis and filtered to remove particulates using a Spin-X centrifuge tube filter (0.45 µm cellulose acetate; Corning Incorporated, Corning, NY). The final dialysis buffer was also filtered (0.2 µm polyethersulfone; Pall Corporation, Ann Arbor, MI) and used for all sample dilutions and as a reference solution for DSC studies.

#### DSC analysis

DSC data were collected with a MicroCal VP-Capillary DSC instrument with integrated autosampler (MicroCal, LLC, Northampton, MA, now part of GE Healthcare). Electrical calibration of the differential power signal and temperature calibration using hydrocarbon temperature standards were performed as part of the manufacturer annual instrument maintenance. Manufacturer performance specifications for the temperature standards are ±0.2°C. Interim instrument performance was assessed using biological standards lysozyme and RNaseA. Manufacturer performance specifications for biological standards are ±0.5°C. Dialyzed plasma samples were diluted 25-fold to obtain a suitable protein concentration for DSC analysis. Samples and dialysate were transferred to 96-well plates and then loaded into the instrument autosampler thermostated at 5°C until analysis. Sample volumes of 400 µL were required to provide sufficient volume for loading of the instrument capillary and ensure proper filling of the 135 µL thermal sensing area. DSC scans were recorded from 20°C to 110°C at a scan rate of 1°C/min with a pre-scan thermostat of 15 minutes, mid feedback mode and a filtering period of 2 seconds. Duplicate DSC scans were obtained for each plasma sample. For each experiment set, we batched samples to ensure the analysis was completed within a seven day window after initial thawing of samples. In developing our experimental procedure we have carefully evaluated sample and buffer scans as a function of run sequence, instrument chamber rinsing and cleaning protocol, and time since preparation. We have examined buffer scans collected at the beginning and end of a sample set and after single or consecutive sample scans and determined acceptable reproducibility and effective cleaning of instrument chambers. We have compared duplicate sample scans collected after a buffer or sample scan and found it is possible to collect consecutive sample scans after extensive rinsing of the instrument chambers with no effect on thermogram profile. We routinely compared duplicate sample scans collected under different run sequences and at different time points to ensure the thermogram profile was reproducible.

DSC data were analyzed using Origin 7 (OriginLab Corporation, Northampton, MA). Raw DSC data were corrected for the instrumental baseline by subtraction of a suitable buffer reference scan. Corrected scans were normalized for the total protein concentration which was determined colorimetrically using the bicinchoninic acid protein assay kit and microplate procedure from Pierce (Pierce, Rockford, IL), with minor modifications to the manufacturer’s protocol. Absorbance readings were taken using a Tecan Sunrise microplate absorbance reader (Tecan U.S., Research Triangle Park, NC). Following normalization, plasma DSC scans were corrected for non-zero baselines by application of a linear baseline fit. Choice of an appropriate sample baseline correction is complicated by the presence of a limited region of post-transition baseline followed by aggregation and precipitation events occurring after the thermal denaturation envelope. We have evaluated all available baseline correction options within the analysis software and found the linear baseline option to give the most consistent results when tested on repeated measurements, different samples and by independent user determinations. We accept that our approach might have limitations but have selected the most consistent baseline correction method that can be applied across all of our studies. Final thermograms were plotted as excess specific heat capacity (cal/°C.g) versus temperature (°C).

#### Statistical analysis of DSC data

Examination of all thermogram data revealed that the temperature range 45–90°C spanned the complete denaturation profile for all samples and scans were truncated to this range for all subsequent analyses. Quantitative comparison of thermograms was achieved through calculation of a number of shape and feature metrics. Parameters considered were total peak area; peak height; peak width at half height; temperature of the peak maximum (T_max_); heat capacity of the primary transition in the range 60–65°C [C_p_^ex^ (Peak 1)]; heat capacity of the secondary transition at 68–72°C [C_p_^ex^ (Peak 2)]; ratio of the first and second transition amplitudes [C_p_^ex^ (Peak 1)]/[C_p_^ex^ (Peak 2)]. Because of the complexity of thermogram shapes, an additional parameter was calculated to provide a measure of the distribution of the area of the denaturation profile relative to the temperature axis. The first moment temperature (T_FM_) was determined according to equation 1:
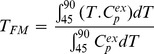
(1)


Thermograms were grouped by pathologic disease status into five groups: healthy controls, LSIL (CIN 1), HSIL (CIN 2, CIN 3), early stage IC (Stage I) and advanced IC (Stage II-IV). Mean thermograms with standard deviations were determined for each study group.

Differences in thermogram shape and feature metrics were assessed by non-parametric methods. First, box charts were used to compare differences in the distributions between each study group. Box charts were constructed with the following form: the bottom and top of the box represented the 25^th^ and 75^th^ percentile, respectively, and the 50th percentile was represented by the band in the middle of the box. The bottom and top ends of the whiskers denoted the 5^th^ and 95^th^ percentiles, respectively. Horizontal lines indicated the minimum and maximum of all the data and the crosses represented the 1^st^ and 99^th^ percentiles. The mean was denoted by an open square. Differences in thermogram metrics for each clinical group were tested for significance using the non-parametric Mann-Whitney U test for unequal medians [34].

### MS Studies

#### Experimental design of MS studies

The order of sample handling and analysis (sample number, order of sample preparation, lyophilization, MALDI-TOF plate spotting, and TOF data acquisition) was randomized to minimize systematic variability during data acquisition. Samples were processed to isolate free peptides (Fraction 1 or FX1), isolate peptides that were bound to total plasma proteins (Fraction 2 or FX2), isolate peptides released during the immunodepletion of albumin and IgG from plasma (Fraction 3 or FX3), and isolate peptides selectively bound to the highly abundant proteins, albumin and IgG (Fraction 4 or FX4). Samples were spotted in triplicate, MALDI-TOF MS data acquired and signal averaged across the three spots. Analysis of averaged data for differentially abundant peptides was conducted using principal component analysis (PCA) and Student’s t-test, followed by manual review of ion intensities for selected peptides. Manual review was performed to ensure that peptides were not excluded as a result of incorrect baseline normalization or baseline subtraction.

#### Sample handling

Samples were thawed once to aliquot into 100 µL volumes for storage at −80°C and once for sample preparation for peptide quantification. Samples were analyzed using two separate aliquots and individual aliquots were analyzed in triplicate. Plasma samples were depleted of albumin and IgG using commercially available affinity spin columns (Sartorius N.A. Inc, Edgewood, NY) according to the manufacturer’s guidelines. Peptides not bound to plasma proteins were isolated using a modification of the precipitation method of Chertov et al [35]. Peptides bound to albumin and IgG were recovered from the albumin/IgG affinity resin by acid stripping with 10% acetic acid. Peptides bound to plasma proteins were recovered from the organic solvent-coagulated proteins by salt or acid ionization. Recovered peptide solutions were lyophilized and resuspended in 0.1% trifluoroacetic acid (TFA) prior to MALDI-TOF MS analysis. Spot urine samples were thawed from −80°C on ice. Urine samples were spun at 1,500×g for 15 minutes at 4°C to pellet cell debris and particulate matter. Urine peptides were isolated using Amicon Ultra-4 (10,000 NMWCO) (Millipore, Billerica, MA) as previously described [26].

#### MS analysis of isolated plasma and urine peptides

All samples were desalted and concentrated for MALDI-TOF MS analysis using C18 ZipTips (Millipore, Billerica, MA). Peptides were eluted from the C18 resin using sample matrix, 5 mg/mL 4-hydroxy-α-cyanocinnamic acid (α-CN), and directly spotted in triplicate onto the MALDI plate. Positive ion MALDI-TOF mass spectra were acquired as described [36]. Peptides selected for further studies were analyzed using the AB4700 in TOF/TOF mode and interpretation of fragmentation data using Mascot (Matrix Science, Boston, MA) ver1.9 as described previously [26].

#### Statistical analysis of MS data

Data files (.t2d) were exported from the AB4700 Proteomics Analyzer and imported into MarkerView software. This software can find spectral peaks by user defined mass tolerance limits or bin spectra via user defined bin sizes. In addition, the user may define minimum and maximum signal responses, which assists in dealing with high-dimensional mass spectral data sets. Data were analyzed using data binning, which allowed for baseline subtraction. Peptide expression data were first examined using PCA. Following data import, the data were preprocessed using no-weighting and either mean-centered or Pareto data scaling prior to PCA. PCA is a method of exploratory data analysis that reduces the complexity of data, while retaining their variability, by transforming the data into a new group of variables, referred to as the principal components. PCA was used to obtain an unbiased assessment of whether or not the MALDI-TOF MS peptide expression data self-sorted into groups corresponding to patients with CIN, cervical cancer and healthy controls without CIN/cervical cancer. Differential peptide and protein expression was compared by unpaired, two-tailed Student’s t-test using aligned MALDI spectra and the averaged peak cluster area for individual peptides. A p-value of <0.05 was considered significant. Differentially expressed peptides were selected for tandem MS analysis using the AB4700 Proteomics Analyzer.

## Results

Plasma specimens from 67 women attending the Division of Gynecologic Oncology clinic and 4 healthy volunteers were collected for DSC analysis. The breakdown of specimen numbers was as follows: control = 4, CIN 1 = 3, CIN 2 = 4, CIN 2–3 = 3; CIN 3 = 22, IC = 35 (FIGO Stage I = 14, II = 10, IIIb = 7, IV = 4); age range = 18–69 years (mean 38.7 years) (Table S1). Complete demographic and clinical information, including biopsy-proven CIN and IC status, was collated for all specimens by members of the clinical study team. Samples provided to the basic science study team were deidentified and devoid of all demographic and clinical information. DSC analysis of plasma samples yielded normalized, baseline-corrected duplicate thermograms for each sample. At this stage, clinical and demographic information was provided to the basic science team for subsequent analyses.

We have previously shown that the thermogram of plasma obtained from healthy individuals is representative of the thermal denaturation of the most abundant plasma proteins weighted according to their average normal plasma concentrations [18]. For the current study, thermograms of plasma obtained from healthy volunteers were averaged to obtain a study-specific ‘healthy control’ profile which accounts for the specific collection and handling protocols employed in the current study. By way of comparison, the previously published mean thermogram was obtained from 15 healthy individuals: 9 males and 6 females (11 African American and 4 Caucasian) ranging in age from 22 to 50 years (mean 37.0 years). The mean thermogram has an area of 5.02±0.23 cal/g and a first moment temperature (T_FM_) of 67.4±0.8°C. The study-specific ‘healthy control’ profile obtained from 4 Caucasian females with a similar age range to the previous study (25–50 years; mean 39.3 years) compares well with similar area (4.94±0.19 cal/g) and T_FM_ (66.3±0.3°C) values.

**Figure 1:**
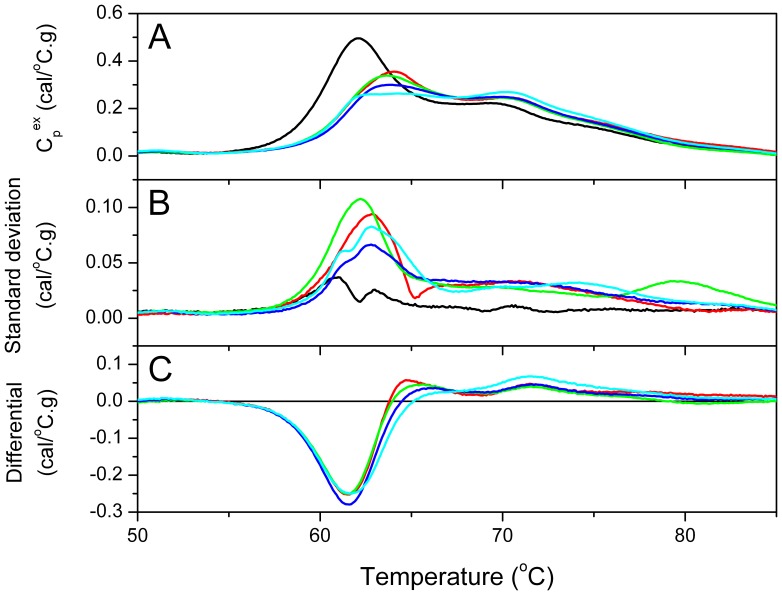
Thermogram profiles associated with progressive cervical disease. Mean thermogram profiles of excess specific heat capacity (C_p_^ex^) versus temperature (Panel A) and standard deviation (Panel B) of each clinical group: controls (black); LSIL (red); HSIL (green); early stage IC [FIGO Stage I] (blue); and advanced IC [FIGO Stage II-IV] (cyan). Thermograms show a progressive shift towards higher denaturation temperatures with increasing disease burden. Difference plots of clinical group thermograms compared to the control group (Panel C) show negative difference peaks ∼62°C and increasing shift and magnitude of higher temperature positive difference peaks. These are hypothesized to reflect the interaction of disease specific components with abundant plasma proteins, principally albumin, with resultant thermal stabilization and alteration of plasma thermogram profiles.

Thermograms were averaged within five clinical groups: control, LSIL, HSIL, Stage I and Stage II-IV. The mean thermograms are distinct from each other and show a progressive change in profile shape (Figure 1A). The main transition in the range 60–65°C becomes progressively lower with an increase in amplitude of the second transition around 70°C. Each clinical group was examined for the effect of patient demographic factors, specifically age, ethnicity, smoking status and parity with no significant effect found on the mean thermograms. The variance associated with each group profile was examined through calculation of standard deviations at each temperature and construction of a plot of standard deviation of heat capacity as a function of temperature (Figure 1B). The healthy control group shows the smallest variance over the entire temperature range. The other clinical groups show higher variance associated primarily with the major transition ∼65°C as well as the second transition ∼70°C and its higher temperature shoulders. The observed thermogram variance is similar to our previous observations [18] and reflects both expected clinical variation in plasma protein levels as well as disease associated protein modifications.

Changes in thermogram profiles associated with disease burden were examined through DSC difference plots which reflect changes in group thermograms relative to the healthy control group (Figure 1C). For each CIN grade or IC stage, there was a negative difference peak centered ∼62°C with higher temperature positive difference peaks. The positive difference peaks shifted to higher temperature and increased in magnitude with increased disease burden. The LSIL group does not follow this trend in positive difference peaks; this could reflect poor definition of this clinical group given the low sample numbers (n = 3) available for evaluation. While these changes in thermogram profile remain under investigation in our laboratory they suggest the thermal stabilization of plasma protein(s) with a denaturation profile appearing ∼62°C. Our previous studies on healthy control samples have shown this thermogram region to be dominated by albumin denaturation and suggest thermal stabilization of albumin in the disease state [18]. This observation could be consistent with stabilizing modifications or interactions with disease components and would be supportive of the interactome hypothesis.

**Figure 2:**
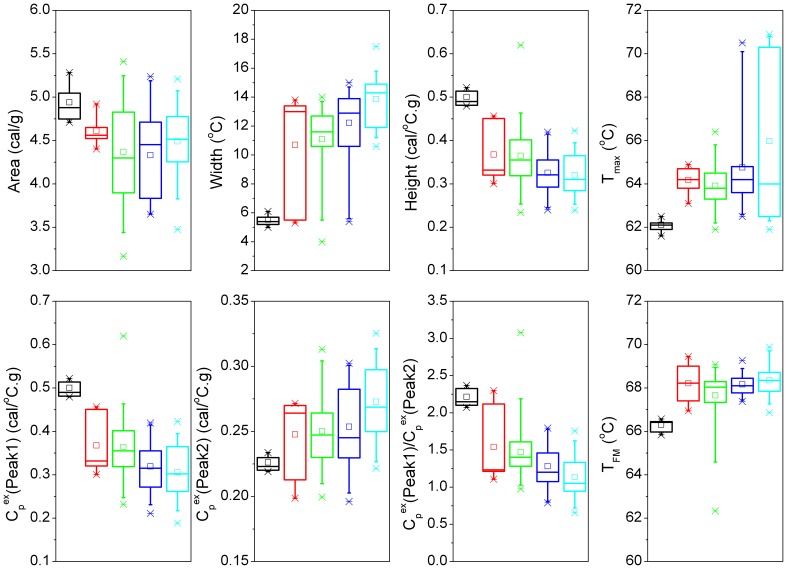
Box charts showing thermogram shape and feature parameters for each clinical group. Trends in thermogram parameters are observed with increasing disease burden from healthy controls (black); LSIL (red); HSIL (green); early stage IC [FIGO Stage I] (blue); and advanced IC [FIGO Stage II-IV] (cyan). Increased width with concomitant decreased height reflects the change in profile shape with disease progression seen in Figure 1A. T_FM_, the first moment temperature corresponding to the geometric center of the thermogram, provided a measure of this shape redistribution. T_FM_ proved superior to T_max_, the temperature of the peak maximum, as a discriminatory metric for advanced IC where large distribution of T_max_ values was correlated with significant variability in the heights of the major profile peaks. Profile width and the ratio of the major transition amplitudes, C_p_^ex^ (Peak 1) and C_p_^ex^ (Peak 2), provided the best metrics for discrimination of clinical status. Summary of statistical analyses of these parameters is shown by Tables 1 and 2. In each box chart the median value is indicated by the horizontal line within the box while the 25^th^ and 75^th^ percentiles are indicated by the lower and upper box edge, respectively. The mean value is indicated by the square within the box. The upper and lower “whiskers” define the 95^th^ and 5^th^ percentiles, respectively. The 99^th^ and 1^st^ percentiles are shown by the crossed symbols and the horizontal lines denoted the minimum and maximum of the data set.

To provide a more quantitative assessment of the observed changes in thermogram with cervical disease status, a comparison was made of the key thermogram parameters (Table 1 and Figure 2). Thermogram profiles are sensitive to changes in solution composition and the stability of components which can be affected by modifications and interactions. As such, the correlation of thermogram profile and their descriptive parameters with clinical status could provide surrogate indicators of disease-associated changes of the plasma proteome. In this vein, we examined thermogram parameters for each clinical group to assess their value as serological disease indicators. Table 1 and Figure 2 show that there is a range of parameter values associated with each clinical group. There is clearly some overlap between groups but clear trends of parameter values are correlated with disease burden. As discussed earlier, the LSIL group is poorly defined by low sample numbers. This group shows significant variation in thermogram parameters and does not follow the general trends exhibited by the other cervical disease groups.

**Table 1:** Thermogram shape and feature parameters for each clinical group.

Parameter	Control [Median (LQ,UQ)]^h^	LSIL^f^ [Median (LQ,UQ)]	HSIL^g^ [Median (LQ,UQ)]	Stage I [Median (LQ,UQ)]	Stage II-IV [Median (LQ,UQ)]
Area (cal/g)	4.89 (4.75,5.05)	4.59 (4.52,4.65)	4.33 (3.90,4.83)	4.46 (3.83,4.71)	4.52 (4.25,4.78)
Width (°C)	5.5 (5.2,5.7)	13.1 (5.5,13.4)	11.7 (10.6,12.7)	13.1 (10.6,13.9)	14.3 (11.9,14.9)
Height (cal/°C.g)	0.50 (0.48,0.51)	0.34 (0.32,0.45)	0.36 (0.32,0.40)	0.32 (0.29,0.36)	0.31 (0.28,0.37)
T_max_ a (°C)	62.2 (61.9,62.2)	64.3 (63.8,64.7)	63.8 (63.3,64.5)	64.3 (63.6,64.8)	64.1 (62.5,70.3)
T_FM_ b (°C)	66.4 (66.0,66.5)	68.3 (67.4,69.0)	68.0 (67.3,68.3)	68.1 (67.8,68.5)	68.4 (67.9,68.7)
C_p_^ex^ (Peak 1)^c^ (cal/°C.g)	0.50 (0.48,0.51)	0.34 (0.32,0.45)	0.36 (0.32,0.40)	0.32 (0.27,0.36)	0.30 (0.26,0.37)
C_p_^ex^ (Peak 2)^d^ (cal/°C.g)	0.23 (0.22,0.23)	0.27 (0.21,0.27)	0.25 (0.23,0.26)	0.25 (0.23,0.28)	0.27 (0.25,0.30)
C_p_^ex^ (Peak 1)/C_p_^ex^ (Peak 2)^e^	2.19 (2.10,2.33)	1.26 (1.21,2.12)	1.40 (1.28,1.61)	1.21 (1.08,1.46)	1.06 (0.95,1.33)

^a^ T_max_, temperature of the peak maximum;

^b^ T_FM_, first moment temperature;

^c^ C_p_^ex^ (Peak 1), excess specific heat capacity of the main transition;

^d^ C_p_^ex^ (Peak 2), excess specific heat capacity of the second transition;

^e^ C_p_^ex^ (Peak 1)/C_p_^ex^ (Peak 2), ratio of the excess specific heat capacities of the main and second transitions;

^f^ LSIL, low-grade squamous intraepithelial lesion (LSIL);

^g^ HSIL, high-grade squamous intraepithelial lesion (LSIL);

^h^ median value, lower quartile and upper quartile.

All eight thermogram parameters showed distinct differences across all disease groups compared with the control group. Thermogram area decreased for all disease groups compared with the control group but showed only small deviations across disease groups. Opposite trends in width and height were observed where width increased concomitant with decreased height associated with disease progression. This width-height compensation with little change in area reflected the redistribution of thermogram area with disease status shown by Figure 1A. T_FM_, the temperature corresponding to the geometric center of the thermogram, provided a measure of the changing distribution of thermogram shape and showed a general increase with disease progression. A wide distribution of T_max_ values was observed for advanced disease groups (Stage II-IV) resulting from variability in the amplitudes of the major transitions. The amplitude of the main transition [C_p_^ex^ (Peak 1)] decreased with disease burden while that of the second transition [C_p_^ex^ (Peak 2)] increased. The ratio of these transitions presented the clearest distinction between disease groups. Differences in thermogram parameter values were tested for statistical significance using the nonparametric Mann-Whitney U test for unequal medians and are summarized in Table 2. Overall, the control group profile was clearly distinct from all disease groups. LSIL did not exhibit statistically significant distinction from the other groups except for the most advanced IC for select thermogram parameters. Again, this might be because of the limited definition of this clinical group. HSIL groups could be distinguished from IC across most thermogram parameters and early stage (Stage I) could also be distinguished from advanced IC (Stage II-IV) by some parameters. Certain thermogram parameters did not display significant sensitivity to clinical status, namely, area and T_max_. T_FM_ was a modestly significant discriminator. The best discrimination was given by profile width and the ratio of the heights of the main and second transitions. Overall profile height was not a good discriminator but heights of the major transitions, and specifically their ratio, provided a good metric for clinical discrimination.

**Table 2:** Statistical comparison of differences in thermogram parameters between clinical groups.

Parameter	Ctrl vs. LSIL^f^	Ctrl vs. HSIL^g^	Ctrl vs. Stg I	Ctrl vs. Stg II-IV	LSIL vs. HSIL	LSIL vs. Stg I	LSIL vs. Stg II-IV	HSIL vs. Stg I	HSIL vs. Stg II-IV	Stg I vs. Stg II-IV
Area (cal/g)	0.0127	0.0087	0.0008	0.0008	0.3972	0.2204	0.4735	0.7021	0.2901	0.2237
Width (°C)	*0.0811*	<0.0001	<0.0001	<0.0001	0.4199	0.3661	0.0257	0.0150	<0.0001	0.0022
Height (cal/°C.g)	0.0007	<0.0001	<0.0001	<0.0001	0.9552	0.2967	*0.0674*	0.0114	0.0007	0.6444
T_max_ a (°C)	0.0024	<0.0001	<0.0001	0.0001	0.3271	0.9820	0.9502	*0.0814*	0.3315	0.9617
T_FM_ b (°C)	0.0007	0.0002	<0.0001	<0.0001	0.2921	0.8430	0.7496	*0.0891*	0.0034	0.2942
C_p_^ex^ (Peak 1)^c^ (cal/°C.g)	0.0007	<0.0001	<0.0001	<0.0001	0.9552	0.2567	0.0358	0.0072	<0.0001	0.3654
C_p_^ex^ (Peak 2)^d^ (cal/°C.g)	0.3450	0.0048	0.0106	<0.0001	0.8136	0.5221	0.1599	0.7857	0.0003	0.0129
C_p_^ex^ (Peak 1)/C_p_^ex^(Peak 2)^e^	0.0293	<0.0001	<0.0001	<0.0001	0.7108	0.2380	0.0496	0.0210	<0.0001	0.0324

^a^ T_max_, temperature of the peak maximum;

^b^ T_FM_, first moment temperature;

^c^ C_p_^ex^ (Peak 1), excess specific heat capacity of the main transition;

^d^ C_p_^ex^ (Peak 2), excess specific heat capacity of the second transition;

^e^ C_p_^ex^ (Peak 1)/C_p_^ex^ (Peak 2), ratio of the excess specific heat capacities of the main and second transitions;

^f^ LSIL, low-grade squamous intraepithelial lesion (LSIL);

^g^ HSIL, high-grade squamous intraepithelial lesion (LSIL); underlined values indicate high confidence of statistically significant differences in median thermogram parameters between clinical groups where p-values were less than 0.05; italics indicate differences in median parameter values which are of moderate statistical significance where p-values were between 0.05 and 0.1.

**Figure 3:**
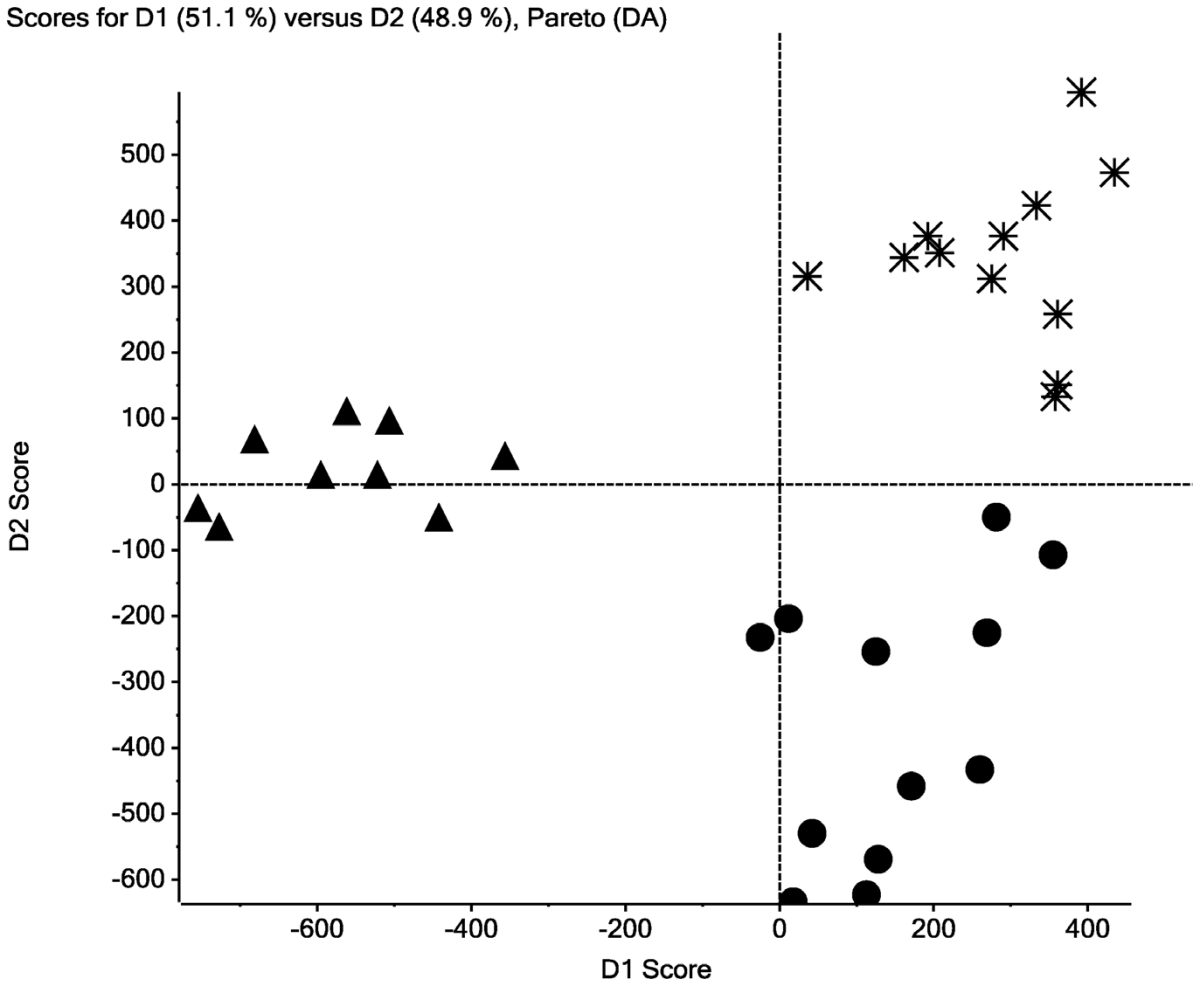
Discriminate principal components analysis (dPCA) of MALDI-TOF MS peptidomic data. MALDI-TOF MS spectra were aligned and binned using Markerview software version 1.2 (Applied Biosystems, Framingham, MA) and analyzed using discriminant Principal Component Analysis (dPCA). The top two principal components from the dPCA analysis of MALDI-TOF MS data for free urine peptides sort cervical cancer (**asterisks**), CIN 2 (**triangles**) and control cervix (**circles**) urine samples into discriminate groups.

**Figure 4:**
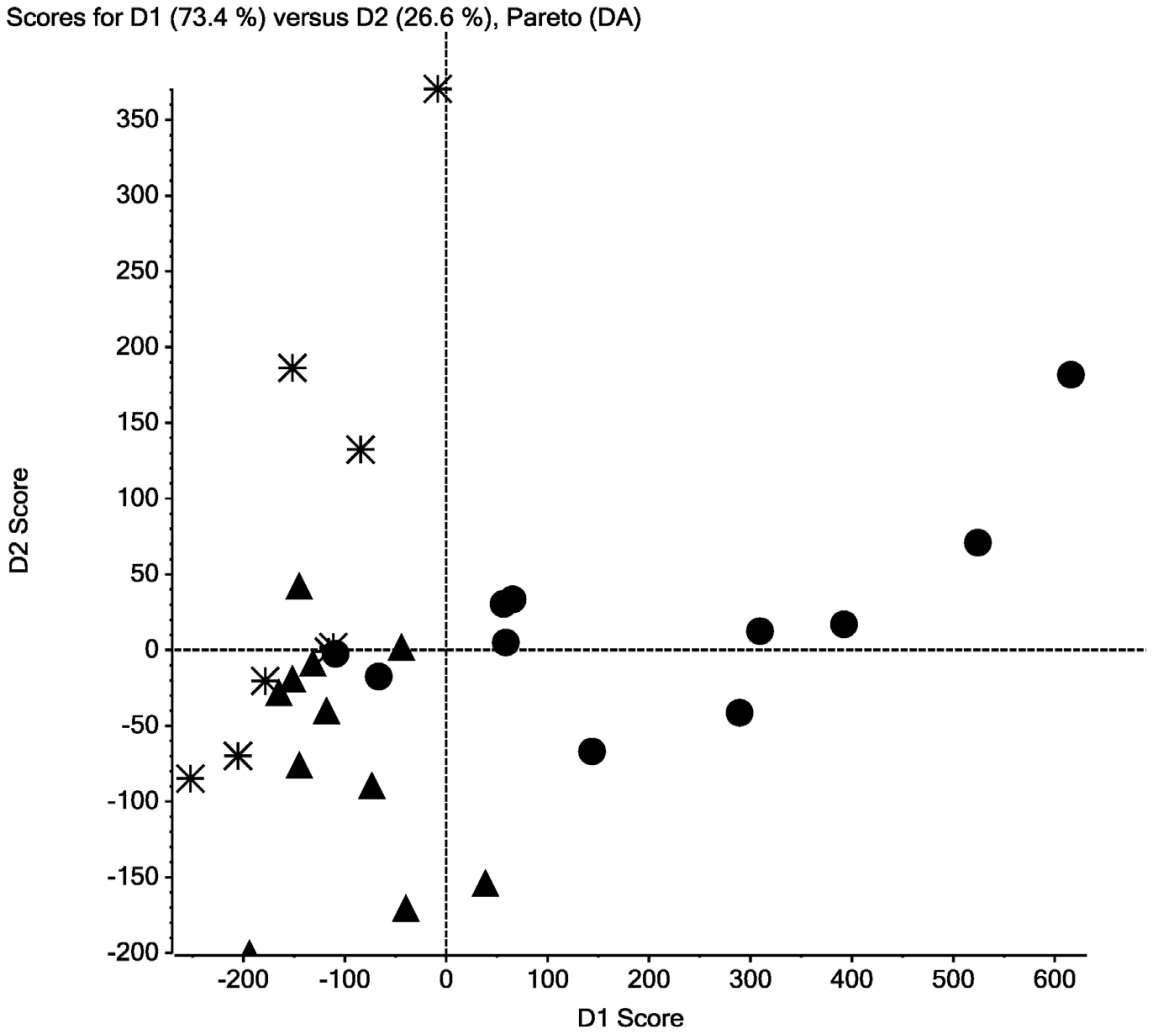
Discriminate principal components analysis (dPCA) of MALDI-TOF MS peptidomic data. MALDI-TOF MS spectra were aligned and binned using Markerview software version 1.2 (Applied Biosystems, Framingham, MA) and analyzed using discriminant Principal Component Analysis (dPCA). The top two principal components from the dPCA analysis of MALDI-TOF MS data for peptides released following precipitation of plasma proteins by addition of acetonitrile/acetic acid. (FX2; Fraction 2) sort cervical cancer (**asterisks**), CIN 2 (**triangles**) and control cervix (**circles**) plasma samples into discriminate groups.

A subset of patient samples analyzed by DSC was selected for MS analysis. Both urine and plasma peptide samples from three groups of patients (four control samples; four CIN 2 samples; four cervical cancer samples) were analyzed using MALDI-TOF MS and computer-assisted methods to compare MS feature data. More peptides were detected collectively within the 12 urine samples (n_urine = _240) than found in the collective serum peptide panels (n_FX1_ = 87; n_FX2_ = 49; n_FX3_ = 75; n_FX4_ = 23). As shown in Figures 3, 4 and S1–3, the peptide expression data self-sorted, suggesting differences in peptide expression between patients with CIN, cervical cancer and patients without CIN 2/cervical cancer (control samples). The discriminate PCA (dPCA or DA) of the urine peptidome and the plasma samples identified the strongest principal components sufficient to sort sample MS data into groups (Figures 3, 4 and S1–3). A total of 15 peptides were differentially abundant at p-values ≤0.05 (Table 3). Using MALDI-TOF/TOF MS methods partial amino acid sequence information was obtained for seven peptides including four peptide fragments of kininogen, two peptide fragments of collagen I-alpha-I and one peptide fragment of collagen XVII-alpha-I. Three kininogen fragments of the light chain of high molecular weight kininogen were present in greater amounts in the FX2 (interactome) fraction of control plasma samples. The largest of these peptides has a sequence of R.KHNLGHGHKHERDQGHGHQ.R. The second and third peptides represent N-terminal shortened peptides of sequences: K.HNLGHGHKHERDQGHGHQ.R and H.NLGHGHKHERDQGHGHQ.R. One kininogen peptide, des-Arg^9^-bradykinin (K.RPPGFSPF.R), was absent in the FX3 fraction of control plasma samples and was equal abundance in the FX3 fractions of cervical cancer and CIN 2 samples. Two collagen 1-alpha-1 fragments (P.GP(OH)P(OH)GPPGPP(OH)GLGG.N and A.GQDGRP(OH)GPP(OH)GPP(OH)G.A) were differentially presenting in the urine increased in CIN 2 over cervical cancer and control samples. One collagen XVII-alpha-1 peptide (E.GLPGPP(OH)GPP(OH)GSFLSN.S) was increased in the urine of CIN 2 patients over cervical cancer patients and absent from control samples.

**Table 3:** Differentially abundant plasma and urine peptides (900–3000 m/z) correlating with cervical cancer, CIN 2 or control samples.

No.	Peptide Mass (m/z)	Sample Set	Protein Identified	Distribution in Samples
1	904.53	FX3^a^	kininogen L,high MW	Absent in Control; Cerv Ca = CIN 2
2	1097.49	Urine	–	CIN 2 = Control>Cerv Ca
3	1126.503	Urine	alpha-1 type I collagen	CIN 2> Cerv Ca >Control
4	1192.67	FX3^a^	–	Absent in Control; Cerv Ca = CIN 2
5	1236.56	Urine	alpha-1 type I collagen	CIN 2> Control>Cerv Ca
6	1425.65	Urine	alpha-1 type XVII collagen	Absent in Control; CIN 2>Cerv Ca
7	1524.71	Urine	–	CIN 2> Cerv Ca = Control
8	1530.91	FX1^b^	–	Absent in CIN 2; Cerv Ca = Control
9	1587.97	Urine	–	CIN 2> Control>Cerv Ca
10	1746.78	FX1^b^, FX2^c^	–	Control>Cerv Ca = CIN 2
11	1943.93	FX2^c^	kininogen L,high MW	Control
12	2080.98	FX2^c^	kininogen L,high MW	Control
13	2209.06	FX2^c^	kininogen L,high MW	Control>Cerv Ca = CIN 2
14	2228.05	FX2^c^	–	Absent in CIN 2; Control>Cerv Ca
15	2271.13	FX2^c^	–	Control>Cerv Ca = CIN 2

^a^ FX3, Fraction 3: peptides released during the immunodepletion of albumin and IgG from plasma.

^b^ FX1, Fraction 1: freely soluble plasma peptides.

^c^ FX2, Fraction 2: peptides bound to total plasma proteins.

## Discussion

This study provided support for the utility of DSC and MS approaches in characterizing cervical disease. Using DSC methodology we have demonstrated distinct thermogram profiles that not only differentiated healthy control subjects from precancerous CIN and IC but also showed a clear distinction between HSIL from both controls and IC and FIGO Stage I IC from more advanced IC (Stage II–IV). The DSC method has value in determining which CIN lesions require treatment and in determining the stage of disease once progression to invasive cancer has taken place. MS analyses identified differentially abundant peptides in both plasma and urine correlated with clinical status. As such, these peptides could serve as surrogate biomarkers of cervical disease.

The pivotal distinction in the management of patients with CIN is between HSIL and LSIL. Accurate identification of HSIL, which carries a higher risk of progression, enables a patient to receive appropriate ablative or excisional surgery whereas those with LSIL can be followed without treatment. Treatment options for IC are dependent on establishing the stage of disease with the important need to determine if the disease is confined to the cervix or has spread locally or regionally. Disease which is confined to the cervix, particularly if 4 cm or less, may be treated with radical surgery, whereas locally advanced disease and greater is treated with chemoradiation. This study examined the utility of DSC in accurately determining cervical disease at the time of patient presentation which could then be used to guide subsequent treatment. DSC thermograms exhibited clear distinction of local disease (FIGO Stage I) from disseminated advanced disease (FIGO Stage II–IV). One downside of our study was the limited availability of CIN 1 (LSIL) clinical specimens and the thermogram for this study group needs further refinement. In addition, CIN 2 (n = 4) and CIN 2–3 (n = 3) had lower representation compared with CIN 3 (n = 22). Thermograms for CIN 2 and CIN 3 were similar in appearance and were grouped together to yield a thermogram for HSIL, an important clinical intervention point. The HSIL thermogram was clearly distinct from both controls and IC suggesting that within the representative sample population included in this study, DSC has potential utility for the identification of HSIL. In order to determine the sensitivity of DSC to discriminate LSIL from HSIL a future study is needed with increased numbers of CIN patient samples representing all grades of CIN.

This study has provided a preliminary demonstration of the diagnostic utility of DSC thermograms in the cervical disease setting. Clear trends in thermogram features are observed between study groups which provide support for the sensitivity of thermograms to disease processes. Differences in sample numbers between groups was not ideal but was dictated by clinical operations. Importantly, the same trends in thermogram parameters were maintained when considering each group as a whole or identical numbers of samples within each group. More work is needed to understand the significance of thermogram profile differences both within and between demographic and clinical groups and this is an area of active investigation in our laboratory. We have observed the sensitivity of thermograms to demographic factors, such as gender and race (unpublished data). Within the current study, the control group displayed the lowest standard deviation of all groups despite have one of the lowest sample numbers (n = 4). This was unrelated to any difference in sample collection protocol which was the same for all control and clinical groups. Demographic variation within the control group was lower than within the clinical groups and this might have contributed to the lower standard deviation. Further, greater variation might be expected within the clinical groups reflective of differences in disease processes or treatment regimens. The development of standardized clinical and analytical procedures and control reference groups for DSC analysis are areas of ongoing work in our laboratory which would enhance the clinical application of DSC.

The basis of the observed changes in thermograms for each clinical group suggests disease specific modulation in the denaturation behavior of the most abundant plasma proteins. We hypothesize that this results from their disease modification or interaction with disease components. One manifestation of this hypothesis is that thermogram changes arise from the interaction of low molecular weight peptides resulting from molecular disease processes. To address these effects, thermogram changes were examined through difference plots for each study group. These showed the shift of denaturating specie(s) centered ∼62°C to higher denaturation temperatures. We have shown previously [18] that the denaturation peak ∼62°C in healthy control samples is dominated by albumin. The changes seen in the difference plots may be consistent with the thermal stabilization of albumin through interaction or modification by disease components which result in the shift of the albumin transition from the unligated temperature of 62°C to higher temperatures. With greater disease progression, increased shifting and magnitude of the positive difference peaks suggest a greater extent of interactions with albumin. The suggestion of disease specific interactions with components of the plasma proteome, that manifests as changes in plasma thermograms, are supportive of the interactome hypothesis but provide no direct evidence. To further investigate the nature of these proposed interactions and provide more direct evidence of the interactome hypothesis, MS peptidomic analyses were employed on a subset of the study samples used for DSC analysis.

MALDI-TOF MS based peptidomic analyses of serum, plasma and urine has previously been demonstrated to have the potential to generate surrogate biomarkers of disease complications [26] and progression of cancer [28], [37], [38], [39], [40]. This study identified a small list of peptides whose abundance was associated with a disease condition. We present here novel data for plasma and urine peptides as surrogate biomarkers of cervical cancer and CIN 2. In these studies urinary collagen I-alpha-I fragments are increased in CIN 2 over cervical cancer over control samples. Additionally a collagen XVII-alpha-I fragment that is increased in CIN 2 over cervical cancer samples is also absent in control samples. The most striking plasma peptidome data supports the interactome theory of peptide portioning to abundant plasma proteins. Here three kininogen light chain fragments proximal to the C-terminus of bradykinin were detected more frequently and in larger amounts in peptides stripped from plasma proteins of control samples. Two of these kininogen peptides (R.KHNLGHGHKHERDQGHGHQ.R and K.HNLGHGHKHERDQGHGHQ.R) have previously been associated with urinary peptidome signatures of prostate and bladder cancer [41]. It is unclear if these reports are correlative to our observation of increased kininogen in plasma.

Taken together, the MS and DSC data support the notion of the differential expression of disease-specific peptides associated with cervical cancer which are observed as changes in plasma thermograms. Although the basis of these changes is open to further investigation, their observation by DSC offers immediate utility in terms of disease detection and monitoring as a complementary approach to existing cervical cancer diagnostic tools. In particular, “spiking” experiments in which the effects of addition of the isolated peptides identified by MS on the thermograms of normal plasma would be a clear extension of this work but is beyond the scope of the current investigation. Regardless of their origin, the thermogram parameters described provide important measures of the statistical significance of differences between disease profiles. DSC and MS offer unique contributions as diagnostic tools. While MS can identify specific peptide biomarkers in plasma, doing so is laborious and time consuming. The advantage of DSC is that it provides, comparatively quickly, a signature thermogram indicative of a disease state. While this signature may lack the fine detail that MS provides, all indications are that the changes in thermograms arise from interactions of the biomarkers with abundant plasma proteins that are sought by MS. MS and DSC are based on different fundamental properties (charge and shape versus thermal stability) and reveal the presence of biomarkers in different ways that are mutually complementary. Thermogram profiles and associated feature parameters could have utility as clinical metrics, in much the same way as current serological analytes, and could provide important complementary measurement of health status and to guide therapeutic assessment.

## Supporting Information

Figure S1 **Discriminate principal components analysis (dPCA) of MALDI-TOF MS peptidomic data.** MALDI-TOF MS spectra were aligned and binned using Markerview software version 1.2 (Applied Biosystems, Framingham, MA) and analyzed using discriminant Principal Component Analysis (dPCA). The top two principal components from the dPCA analysis of MALDI-TOF MS data for free plasma peptides (Fraction 1 or FX1) sort cervical cancer (**asterisks**), CIN 2 (**triangles**) and control cervix (**circles**) plasma samples into discriminate groups.
(TIF)

Figure S2 **Discriminate principal components analysis (dPCA) of MALDI-TOF MS peptidomic data.** MALDI-TOF MS spectra were aligned and binned using Markerview software version 1.2 (Applied Biosystems, Framingham, MA) and analyzed using discriminant Principal Component Analysis (dPCA). The top two principal components from the dPCA analysis of MALDI-TOF MS data for peptides released during the immunodepletion of albumin and IgG from plasma (Fraction 3 or FX3) sort cervical cancer (**asterisks**), CIN 2 (**triangles**) and control cervix (**circles**) plasma samples into discriminate groups.
(TIF)

Figure S3 **Discriminate principal components analysis (dPCA) of MALDI-TOF MS peptidomic data.** MALDI-TOF MS spectra were aligned and binned using Markerview software version 1.2 (Applied Biosystems, Framingham, MA) and analyzed using discriminant Principal Component Analysis (dPCA). The top two principal components from the dPCA analysis of MALDI-TOF MS data for peptides selectively bound to the highly abundant proteins, albumin and IgG (Fraction 4 or FX4) sort cervical cancer (**asterisks**), CIN 2 (**triangles**) and control cervix (**circles**) plasma samples into discriminate groups.
(TIF)

Table S1 **Demographic and clinical characteristics of the study population.**
(DOCX)
