# Supplementary material for: Detection of Cervical Cancer Biomarker Patterns in Blood Plasma and Urine by Differential Scanning Calorimetry and Mass Spectrometry
Source: PLoS One. 2014 Jan 8;9(1):e84710. doi: 10.1371/journal.pone.0084710 (PMC3885574; doi:10.1371/journal.pone.0084710)
Supplement: Table S1 — Demographic and clinical characteristics of the study population. (DOCX) [file pone.0084710.s004.docx]

**Table S1. Demographic and clinical characteristics of the study population**

| **Characteristic** | **No. of patients** | **%** |
| --- | --- | --- |
| Total number included in the study | 71 |  |
| **Age, years** | | |
| 18-29 | 22 | 31.0 |
| 30-39 | 17 | 23.9 |
| 40-49 | 16 | 22.5 |
| 50-59 | 6 | 8.5 |
| 60-69 | 10 | 14.1 |
| **Ethnicity** | | |
| Caucausian | 56 | 78.9 |
| African American | 9 | 12.7 |
| Vietnamese | 1 | 1.4 |
| Hispanic | 3 | 4.2 |
| Asian | 2 | 2.8 |
| **Smoking status** | | |
| Smoker | 38 | 53.5 |
| Non-smoker | 24 | 33.8 |
| Unknown | 9 | 12.7 |
| **Parity** | | |
| 0 | 7 | 9.9 |
| 1 | 10 | 14.1 |
| 2 | 23 | 32.4 |
| 3 | 7 | 9.9 |
| 4 | 9 | 12.7 |
| ≥ 5 | 4 | 5.6 |
| Unknown | 11 | 15.5 |
| **Grade/Stage** | | |
| No evidence of disease | 4 | 5.6 |
| CIN 1 | 3 | 4.2 |
| CIN 2 | 4 | 5.6 |
| CIN 3 | 25 | 35.2 |
| Stage I | 14 | 19.7 |
| Stage II | 10 | 14.1 |
| Stage IIIb/IV | 11 | 15.5 |
| **Histology subtype** | | |
| No evidence of disease | 4 | 5.6 |
| CIN 1 | 3 | 4.2 |
| CIN 2 | 4 | 5.6 |
| CIN 2-3 | 3 | 4.2 |
| CIN 3 | 21 | 29.6 |
| Squamous cell carcinoma | 30 | 42.3 |
| Adenosquamous carcinoma | 1 | 1.4 |
| Adenocarcinoma | 3 | 4.2 |
| Neuroendocrine carcinoma | 1 | 1.4 |
| Metastatic | 1 | 1.4 |
| **Regional lymph node status** | | |
| Not applicable (control or CIN) | 36 | 50.7 |
| No regional lymph node metastasis | 11 | 15.5 |
| Regional lymph node metastasis | 11 | 15.5 |
| Cannot be assessed/Unknown | 13 | 18.3 |
| **Distant metastasis status** | | |
| Not applicable (control or CIN) | 36 | 50.7 |
| Distant metastasis | 1 | 1.4 |
| No distant metastasis | 20 | 28.2 |
| Cannot be assessed/Unknown | 14 | 19.7 |
